# Observational and genetic associations between cardiorespiratory fitness and cancer: a UK Biobank and international consortia study

**DOI:** 10.1038/s41416-023-02489-3

**Published:** 2023-12-06

**Authors:** Eleanor L. Watts, Tomas I. Gonzales, Tessa Strain, Pedro F. Saint-Maurice, D. Timothy Bishop, Stephen J. Chanock, Mattias Johansson, Temitope O. Keku, Loic Le Marchand, Victor Moreno, Polly A. Newcomb, Christina C. Newton, Rish K. Pai, Mark P. Purdue, Cornelia M. Ulrich, Karl Smith-Byrne, Bethany Van Guelpen, The PRACTICAL consortium, CRUK, BPC3, CAPS, PEGASUS, Rosalind A. Eeles, Christopher A. Haiman, Zsofia Kote-Jarai, Fredrick R. Schumacher, Sara Benlloch, Ali Amin Al Olama, Kenneth R. Muir, Sonja I. Berndt, David V. Conti, Fredrik Wiklund, Stephen J. Chanock, Ying Wang, Catherine M. Tangen, Jyotsna Batra, Judith A. Clements, Henrik Grönberg, Nora Pashayan, Johanna Schleutker, Demetrius Albanes, Stephanie J. Weinstein, Alicja Wolk, Catharine M. L. West, Lorelei A. Mucci, Géraldine Cancel-Tassin, Stella Koutros, Karina Dalsgaard Sørensen, Eli Marie Grindedal, David E. Neal, Freddie C. Hamdy, Jenny L. Donovan, Ruth C. Travis, Robert J. Hamilton, Sue Ann Ingles, Barry S. Rosenstein, Yong-Jie Lu, Graham G. Giles, Robert J. MacInnis, Adam S. Kibel, Ana Vega, Manolis Kogevinas, Kathryn L. Penney, Jong Y. Park, Janet L. Stanford, Cezary Cybulski, Børge G. Nordestgaard, Sune F. Nielsen, Hermann Brenner, Christiane Maier, Jeri Kim, Esther M. John, Manuel R. Teixeira, Susan L. Neuhausen, Kim De Ruyck, Azad Razack, Lisa F. Newcomb, Davor Lessel, Radka Kaneva, Nawaid Usmani, Frank Claessens, Paul A. Townsend, Jose Esteban Castelao, Monique J. Roobol, Florence Menegaux, Kay-Tee Khaw, Lisa Cannon-Albright, Hardev Pandha, Stephen N. Thibodeau, David J. Hunter, Peter Kraft, William J. Blot, Elio Riboli, Felix R. Day, Katrien Wijndaele, Nicholas J. Wareham, Charles E. Matthews, Steven C. Moore, Soren Brage

**Affiliations:** 1 Division of Cancer Epidemiology and Genetics, National Cancer Institute, National Institutes of Health, Rockville, MD USA; 2 MRC Epidemiology Unit, School of Clinical Medicine, Institute of Metabolic Science, University of Cambridge, Cambridge, UK; 3 Leeds Institute of Cancer and Pathology, University of Leeds, Leeds, UK; 4 Genomics Branch, International Agency for Research on Cancer, World Health Organization, Lyon, France; 5 Center for Gastrointestinal Biology and Disease, University of North Carolina, Chapel Hill, NC USA; 6 University of Hawaii Cancer Center, Honolulu, HI USA; 7 Oncology Data Analytics Program, Catalan Institute of Oncology-IDIBELL, L’Hospitalet de Llobregat, Barcelona, Spain; 8 CIBER Epidemiología y Salud Pública (CIBERESP), Madrid, Spain; 9 Department of Clinical Sciences, Faculty of Medicine and University of Barcelona Institute for Complex Systems (UBICS), University of Barcelona, Barcelona, Spain; 10 ONCOBEL Program, Bellvitge Biomedical Research Institute (IDIBELL), L’Hospitalet de Llobregat, Barcelona, Spain; 11 Public Health Sciences Division, Fred Hutchinson Cancer Research Center, Seattle, WA USA; 12 School of Public Health, University of Washington, Seattle, WA USA; 13 Department of Population Science, American Cancer Society, Atlanta, GA USA; 14 Department of Laboratory Medicine and Pathology, Mayo Clinic Arizona, Scottsdale, AZ USA; 15 Huntsman Cancer Institute and Department of Population Health Sciences, University of Utah, Salt Lake City, UT USA; 16 Nuffield Department of Population Health, University of Oxford, Oxford, UK; 17 Department of Radiation Sciences, Oncology Unit, Umeå University, Umeå, Sweden; 18 Wallenberg Centre for Molecular Medicine, Umeå University, Umeå, Sweden; 19 The Institute of Cancer Research, London, SM2 5NG UK; 20 Royal Marsden NHS Foundation Trust, London, SW3 6JJ UK; 21 Center for Genetic Epidemiology, Department of Preventive Medicine, Keck School of Medicine, University of Southern California/Norris Comprehensive Cancer Center, Los Angeles, CA 90015 USA; 22 Department of Population and Quantitative Health Sciences, Case Western Reserve University, Cleveland, OH 44106-7219 USA; 23 Seidman Cancer Center, University Hospitals, Cleveland, OH 44106 USA; 24 Centre for Cancer Genetic Epidemiology, Department of Public Health and Primary Care, University of Cambridge, Strangeways Research Laboratory, Cambridge, CB1 8RN UK; 25 University of Cambridge, Department of Clinical Neurosciences, Stroke Research Group, R3, Box 83, Cambridge Biomedical Campus, Cambridge, CB2 0QQ UK; 26 Division of Population Health, Health Services Research and Primary Care, University of Manchester, Oxford Road, Manchester, M13 9PL UK; 27 Division of Cancer Epidemiology and Genetics, National Cancer Institute, NIH, Bethesda, MD 20892 USA; 28 Department of Medical Epidemiology and Biostatistics, Karolinska Institute, SE-171 77 Stockholm, Sweden; 29 Department of Population Science, American Cancer Society, 250 Williams Street, Atlanta, GA 30303 USA; 30 SWOG Statistical Center, Fred Hutchinson Cancer Research Center, Seattle, WA 98109 USA; 31 Translational Research Institute, Brisbane, QLD 4102 Australia; 32 Australian Prostate Cancer Research Centre-Qld, Institute of Health and Biomedical Innovation and School of Biomedical Sciences, Queensland University of Technology, Brisbane, QLD 4059 Australia; 33 Australian Prostate Cancer Research Centre-Qld, Queensland University of Technology, Brisbane, QLD Australia; 34 Prostate Cancer Research Program, Monash University, Melbourne, VIC Australia; 35 Dame Roma Mitchell Cancer Centre, University of Adelaide, Adelaide, SA Australia; 36 Chris O’Brien Lifehouse and The Kinghorn Cancer Centre, Sydney, NSW Australia; 37 Department of Applied Health Research, University College London, London, WC1E 7HB UK; 38 Centre for Cancer Genetic Epidemiology, Department of Oncology, University of Cambridge, Strangeways Laboratory, Worts Causeway, Cambridge, CB1 8RN UK; 39 Institute of Biomedicine, University of Turku, Turku, Finland; 40 Department of Medical Genetics, Genomics, Laboratory Division, Turku University Hospital, PO Box 52, 20521 Turku, Finland; 41 Department of Surgical Sciences, Uppsala University, 75185 Uppsala, Sweden; 42 Division of Cancer Sciences, University of Manchester, Manchester Academic Health Science Centre, Radiotherapy Related Research, The Christie Hospital NHS Foundation Trust, Manchester, M13 9PL UK; 43 Department of Epidemiology, Harvard T. H. Chan School of Public Health, Boston, MA 02115 USA; 44 CeRePP, Tenon Hospital, F-75020 Paris, France; 45 Sorbonne Universite, GRC n° 5, AP-HP, Tenon Hospital, 4 rue de la Chine, F-75020 Paris, France; 46 Department of Molecular Medicine, Aarhus University Hospital, Palle Juul-Jensen Boulevard 99, 8200 Aarhus N, Denmark; 47 Department of Clinical Medicine, Aarhus University, DK-8200 Aarhus N, Denmark; 48 Department of Medical Genetics, Oslo University Hospital, 0424 Oslo, Norway; 49 Nuffield Department of Surgical Sciences, University of Oxford, Room 6603, Level 6, John Radcliffe Hospital, Headley Way, Headington, Oxford, OX3 9DU UK; 50 University of Cambridge, Department of Oncology, Box 279, Addenbrooke’s Hospital, Hills Road, Cambridge, CB2 0QQ UK; 51 Cancer Research UK, Cambridge Research Institute, Li Ka Shing Centre, Cambridge, CB2 0RE UK; 52 Nuffield Department of Surgical Sciences, University of Oxford, Oxford, OX1 2JD UK; 53 Faculty of Medical Science, University of Oxford, John Radcliffe Hospital, Oxford, UK; 54 Population Health Sciences, Bristol Medical School, University of Bristol, Bristol, BS8 2PS UK; 55 Department. of Surgical Oncology, Princess Margaret Cancer Centre, Toronto, ON M5G 2M9 Canada; 56 Department. of Surgery (Urology), University of Toronto, Toronto, ON Canada; 57 Department of Preventive Medicine, Keck School of Medicine, University of Southern California/Norris Comprehensive Cancer Center, Los Angeles, CA 90015 USA; 58 Department of Radiation Oncology and Department of Genetics and Genomic Sciences, Box 1236, Icahn School of Medicine at Mount Sinai, One Gustave L. Levy Place, New York, NY 10029 USA; 59 Centre for Cancer Biomarker and Biotherapeutics, Barts Cancer Institute, Queen Mary University of London, John Vane Science Centre, Charterhouse Square, London, EC1M 6BQ UK; 60 Cancer Epidemiology Division, Cancer Council Victoria, 615 St Kilda Road, Melbourne, VIC 3004 Australia; 61 Centre for Epidemiology and Biostatistics, Melbourne School of Population and Global Health, The University of Melbourne, Grattan Street, Parkville, VIC 3010 Australia; 62 Precision Medicine, School of Clinical Sciences at Monash Health, Monash University, Clayton, VIC 3168 Australia; 63 Division of Urologic Surgery, Brigham and Women’s Hospital, 75 Francis Street, Boston, MA 02115 USA; 64 Fundación Pública Galega Medicina Xenómica, Santiago de Compostela, Compostela, 15706 Spain; 65 Instituto de Investigación Sanitaria de Santiago de Compostela, Santiago de Compostela, Compostela, 15706 Spain; 66 Centro de Investigación en Red de Enfermedades Raras (CIBERER), Barcelona, Spain; 67 ISGlobal, Barcelona, Spain; 68 IMIM (Hospital del Mar Medical Research Institute), Barcelona, Spain; 69 Universitat Pompeu Fabra (UPF), Barcelona, Spain; 70 CIBER Epidemiología y Salud Pública (CIBERESP), 28029 Madrid, Spain; 71 Channing Division of Network Medicine, Department of Medicine, Brigham and Women’s Hospital/Harvard Medical School, Boston, MA 02115 USA; 72 Department of Cancer Epidemiology, Moffitt Cancer Center, 12902 Magnolia Drive, Tampa, FL 33612 USA; 73 Division of Public Health Sciences, Fred Hutchinson Cancer Research Center, Seattle, WA 98109-1024 USA; 74 Department of Epidemiology, School of Public Health, University of Washington, Seattle, WA 98195 USA; 75 International Hereditary Cancer Center, Department of Genetics and Pathology, Pomeranian Medical University, 70-115 Szczecin, Poland; 76 Faculty of Health and Medical Sciences, University of Copenhagen, 2200 Copenhagen, Denmark; 77 Department of Clinical Biochemistry, Herlev and Gentofte Hospital, Copenhagen University Hospital, Herlev, 2200 Copenhagen Denmark; 78 Division of Clinical Epidemiology and Aging Research, German Cancer Research Center (DKFZ), D-69120 Heidelberg, Germany; 79 German Cancer Consortium (DKTK), German Cancer Research Center (DKFZ), D-69120 Heidelberg, Germany; 80 Division of Preventive Oncology, German Cancer Research Center (DKFZ) and National Center for Tumor Diseases (NCT), Im Neuenheimer Feld 460, 69120 Heidelberg, Germany; 81 Humangenetik Tuebingen, Paul-Ehrlich-Str 23, D-72076 Tuebingen, Germany; 82 The University of Texas M. D. Anderson Cancer Center, Department of Genitourinary Medical Oncology, 1515 Holcombe Blvd., Houston, TX 77030 USA; 83 Departments of Epidemiology & Population Health and of Medicine, Division of Oncology, Stanford Cancer Institute, Stanford University School of Medicine, Stanford, CA 94304 USA; 84 Department of Laboratory Genetics, Portuguese Oncology Institute of Porto (IPO Porto) / Porto Comprehensive Cancer Center, Porto, Portugal; 85 Cancer Genetics Group, IPO Porto Research Center (CI-IPOP) / RISE@CI-IPOP (Health Research Network), Portuguese Oncology Institute of Porto (IPO Porto) / Porto Comprehensive Cancer Center, Porto, Portugal; 86 School of Medicine and Biomedical Sciences (ICBAS), University of Porto, Porto, Portugal; 87 Department of Population Sciences, Beckman Research Institute of the City of Hope, 1500 East Duarte Road, Duarte, CA 91010 USA; 88 Ghent University, Faculty of Medicine and Health Sciences, Basic Medical Sciences, Proeftuinstraat 86, B-9000 Gent, Belgium; 89 Department of Surgery, Faculty of Medicine, University of Malaya, 50603 Kuala Lumpur, Malaysia; 90 Department of Urology, University of Washington, 1959 NE Pacific Street, Box 356510, Seattle, WA 98195 USA; 91 Institute of Human Genetics, University Medical Center Hamburg-Eppendorf, D-20246 Hamburg, Germany; 92 Molecular Medicine Center, Department of Medical Chemistry and Biochemistry, Medical University of Sofia, Sofia, 2 Zdrave Str, 1431 Sofia, Bulgaria; 93 Department of Oncology, Cross Cancer Institute, University of Alberta, 11560 University Avenue, Edmonton, AB T6G 1Z2 Canada; 94 Division of Radiation Oncology, Cross Cancer Institute, 11560 University Avenue, Edmonton, AB T6G 1Z2 Canada; 95 Molecular Endocrinology Laboratory, Department of Cellular and Molecular Medicine, KU Leuven, Leuven, BE-3000 Belgium; 96 Division of Cancer Sciences, Manchester Cancer Research Centre, Faculty of Biology, Medicine and Health, Manchester Academic Health Science Centre, NIHR Manchester Biomedical Research Centre, Health Innovation Manchester, Univeristy of Manchester, Manchester, M13 9WL UK; 97 The University of Surrey, Guildford, Surrey GU2 7XH UK; 98 Genetic Oncology Unit, CHUVI Hospital, Complexo Hospitalario Universitario de Vigo, Instituto de Investigación Biomédica Galicia Sur (IISGS), 36204 Vigo (Pontevedra), Spain; 99 Department of Urology, Erasmus University Medical Center, Cancer Institute, 3015 GD Rotterdam, The Netherlands; 100 “Exposome and Heredity”, CESP (UMR 1018), Faculté de Médecine, Université Paris-Saclay, Inserm, Gustave Roussy, Villejuif, France; 101 Clinical Gerontology Unit, University of Cambridge, Cambridge, CB2 2QQ UK; 102 Division of Epidemiology, Department of Internal Medicine, University of Utah School of Medicine, Salt Lake City, UT 84132 USA; 103 George E. Wahlen Department of Veterans Affairs Medical Center, Salt Lake City, UT 84148 USA; 104 Department of Laboratory Medicine and Pathology, Mayo Clinic, Rochester, MN 55905 USA; 105 Program in Genetic Epidemiology and Statistical Genetics, Department of Epidemiology, Harvard School of Public Health, Boston, MA USA; 106 Division of Epidemiology, Department of Medicine, Vanderbilt University Medical Center, 2525 West End Avenue, Suite 800, Nashville, TN 37232 USA; 107 International Epidemiology Institute, Rockville, MD 20850 USA; 108 Department of Epidemiology and Biostatistics, School of Public Health, Imperial College London, London, SW7 2AZ UK

**Keywords:** Risk factors, Cancer epidemiology

## Abstract

**Background:**

The association of fitness with cancer risk is not clear.

**Methods:**

We used Cox proportional hazards models to estimate hazard ratios (HRs) and 95% confidence intervals (CIs) for risk of lung, colorectal, endometrial, breast, and prostate cancer in a subset of UK Biobank participants who completed a submaximal fitness test in 2009-12 (*N* = 72,572). We also investigated relationships using two-sample Mendelian randomisation (MR), odds ratios (ORs) were estimated using the inverse-variance weighted method.

**Results:**

After a median of 11 years of follow-up, 4290 cancers of interest were diagnosed. A 3.5 ml O_2_⋅min^−1^⋅kg^−1^ total-body mass increase in fitness (equivalent to 1 metabolic equivalent of task (MET), approximately 0.5 standard deviation (SD)) was associated with lower risks of endometrial (HR = 0.81, 95% CI: 0.73–0.89), colorectal (0.94, 0.90–0.99), and breast cancer (0.96, 0.92–0.99). In MR analyses, a 0.5 SD increase in genetically predicted O_2_⋅min^−1^⋅kg^−1^ fat-free mass was associated with a lower risk of breast cancer (OR = 0.92, 95% CI: 0.86–0.98). After adjusting for adiposity, both the observational and genetic associations were attenuated.

**Discussion:**

Higher fitness levels may reduce risks of endometrial, colorectal, and breast cancer, though relationships with adiposity are complex and may mediate these relationships. Increasing fitness, including via changes in body composition, may be an effective strategy for cancer prevention.

## Introduction

Until recently epidemiological studies have largely focused on the role of physical activity behaviours with cancer risk [1]. Cardiorespiratory fitness (referred to here as ‘fitness’) is distinct from physical activity as it describes the capacity of the circulatory and respiratory systems to supply oxygen to skeletal muscle during prolonged physical activity [2, 3]. Fitness is generally objectively measured and has a stronger genetic component than habitual physical activity [2–4].

Higher fitness is associated with good cardiometabolic health, including lower visceral adipose tissue, inflammation and insulin sensitivity, and may, therefore, reduce the risk of cancer [5–8]. Previous studies report that people with higher fitness have lower risks of all-cause mortality, cancer mortality and cardiovascular disease [5, 9–11], but the relationship between fitness and incident cancers are less clear. Some studies have reported inverse associations between fitness and lung and colorectal cancers [12–17], while for prostate cancer associations have been reported to be null or positive [13–15, 17–20]. Only one prior study has investigated associations between fitness and female-specific incident cancers, and did not find evidence of a relationship [14].

A limitation of observational epidemiological studies includes the possibility of residual confounding and reverse causation. Mendelian randomisation (MR) uses germline genetic variants as proxies of biological traits to generate instrumental variables and estimate their associations with disease risk. Because germline genetic variants are fixed and randomly allocated at conception, this technique may be less likely to be affected by biases and confounding factors (such as preclinical disease and smoking history). This is the first study to use MR to investigate fitness and cancer risk.

We aimed to assess the associations of measured fitness and risk of common cancers (lung, colon, rectal, endometrial, female breast, and prostate cancer) using observational methods in the UK Biobank. In secondary analyses, we used a two-sample MR framework, using genetically predicted fitness, as an instrumental variable derived from UK Biobank [21] and genetic case control data from consortia for those same sites, plus pancreatic cancer and renal cell carcinoma for which observational analyses in the UK Biobank are underpowered. By integrating evidence from both observational epidemiology and MR approaches, we aim to strengthen the basis for causal inference [22].

## Methods

### UK Biobank study population

The UK Biobank study is a population-based prospective cohort study of 502,625 adults aged 40 to 69 years. A description of the study protocol is available online [23]. Participants were registered with the UK National Health Service and lived within 40 km of a UK Biobank assessment centre in England, Wales, and Scotland. Baseline data were collected between 2006 and 2010. A repeat-measures substudy was conducted between 2012 and 2013.

### UK Biobank cardiorespiratory fitness assessment

An individualised submaximal cycle ergometer test was implemented in 2009 and offered to 75,087 participants during baseline data collection, 17,109 participants during the repeat assessment study, and 2877 participants at both timepoints; 97,950 tests were offered in total. For those participants who were offered a test at both timepoints, the earliest fitness test completed by the participant was used to maximise follow-up duration. Participant baseline data were collected on the same day as their exercise test. The test was individualised to each participant’s exercise capacity and risk level for engaging in exercise. Participants with lower exercise capacity or higher risk for exercise-related complications were offered a test with lower work rates, while those with higher exercise capacity or lower risk were offered a test with higher work rates. A description of the exercise test individualisation process and maximal oxygen consumption (VO_2_ max; ml O_2_⋅min^−1^⋅kg^−1^) estimation process is provided in Supplementary Methods; the test protocol is available online [24]. VO_2_ max was estimated in two ways: scaled by total-body mass (VO_2_max_tbm_ [3.5 ml O_2_⋅min^−1^⋅kg^−1^ total-body mass=1 MET]) and scaled by fat-free mass (VO_2_max_ffm_) [25, 26]. VO_2_max_ffm_ represents the ability of skeletal muscle to use oxygen during maximal exercise, whereas VO_2_max_tbm_ is more representative of aerobic performance capacity [27].

#### Genetic instrument for cardiorespiratory fitness

Full details of the fitness genome-wide association study (GWAS) are available elsewhere [21]. In brief, single nucleotide polymorphisms (SNPs) associated with fitness were identified from a GWAS based on UK Biobank participants of European ancestry who participated in the fitness test (*N* included = 69,416). Fitness was estimated using the same framework method described above, scaled by fat-free mass and using resting heart rate data from the full cohort, excluding those taking beta-blockers (*N* included = 452,941) (*P* < 5 × 10^−8^ significance threshold).

The Radial plot method was used to select eligible resting heart-rate associated genetic variants for fitness by removing heterogeneous outliers for the genetic variants, of which 149 were also nominally significant in the fitness GWAS (*p* < 0.05) [28]. The genetic instrument for fitness included 14 fitness and 149 fitness and resting heart rate variants with prioritisation given to the variants identified in the fitness GWAS. In total, 160 independent (r² > 0.01) genetic variants were included in our instrument for fitness [21].

### Cancer ascertainment

#### Observational analysis

Cancer registration data were provided via record linkage to national cancer and death registries, until the following censoring dates: 31 July 2019 in England and Wales and 31 October 2015 in Scotland. Cancers occurring after the registry censoring dates were identified using Hospital Episode Statistics (HES), until the following censoring dates: 30 September 2021 in England, 31 July 2021 in Scotland and 28 February 2018 in Wales (see Supplementary Methods for cancer site definitions).

Of the 84,792 fitness tests analysed after preliminary exclusions (i.e., participant withdrawal of data, ‘high risk’ for exercise; see Supplementary Fig. 1), we retained a preliminary analytic sample of 79,347 participants after additionally excluding 3209 participants for missing data, 1017 due to test data quality, 1219 with missing weight, fat-free mass, or heart rate, and 44 for whom fitness estimation could not be applied. We then excluded 5180 participants with prevalent cancer at baseline and 1551 participants diagnosed with cancer within two years of follow-up. The final analytic sample was 72,572 participants. Health and sociodemographic characteristics were described across age-adjusted and sex-specific fitness tertiles [29].

#### Genetic cancer data

Risk estimates may be biased when instrumental variables and outcomes are identified from the same sample [30]. We, therefore, used independent GWAS data from international consortia. This includes breast (including estrogen receptor (ER)+ and ER− subtypes) [31, 32], prostate (including aggressive disease) [33], endometrial [34], ovarian [35], lung (including for never smokers) [36], and colorectal cancer (including colon, rectal, male colorectal and female colorectal, distal colon and proximal colon) [37, 38]. We also included pancreatic cancer and renal cell carcinoma [39–42]. Included sites and subtypes were chosen based on data availability. Further information for the genetic case control studies is available in Supplementary Table 1.

### Statistical analysis

#### Observational analysis

Cox regression models with age as the underlying timescale were used to estimate hazard ratios (HRs) and 95% confidence intervals (CIs) per 3.5 ml⋅O_2_⋅min^−1^⋅kg^−1^ total-body mass and 5.0 ml⋅O_2_⋅min^−1^⋅kg^−1^ fat-free mass for risk of cancer diagnosis. Models were adjusted for possible confounding factors and for female reproductive cancers (breast, endometrial and ovarian cancers) we additionally adjusted for reproductive factors (see Supplementary Methods). Multivariate imputation by chained equations was used to impute missing covariate values.

Adiposity may partially mediate and confound the relationship between fitness and cancer risk (Supplementary Fig. 2). Therefore, we evaluated the role of adiposity in fitness-to-cancer associations both with and without adjustment for either BMI (for models with VO_2_max scaled by total-body mass) or fat mass (for models with VO_2_max scaled by fat-free mass).

We have shown previously that repeat assessments of the UK Biobank fitness test will elicit moderately stable fitness estimates (regression dilution ratio = 0.79, standard error = 0.01) [43]. This source of measurement error will influence the strength of observed health associations. Therefore, in a sensitivity analysis, we also provide regression dilution calibrated estimates of fitness-to-cancer associations using established statistical techniques [44]. The shape of dose-response relationships between fitness and risk of cancer diagnosis was investigated using cubic spline regression models. Each model used two knots placed at the 33rd and 67th percentile of the fitness distribution. Reference values were set to the mean fitness value for each specific analysis (see Supplementary Figs. 3 and 4).

##### Sensitivity analysis

Subgroup analyses for colorectal cancer were examined by sex, and associations for fitness and lung cancer were re-examined after restricting the analysis to never-smokers only. Subgroups were chosen a priori on the basis of data availability and previous evidence for heterogeneity in the associations [1]. We also included a minimally adjusted model to investigate the influence of mediators and/or confounders.

### Mendelian randomisation

The MR estimation for fitness and cancer was conducted using the inverse-variance weighted (IVW) method [45]. We additionally calculated the I^2^_GX_ statistic to assess measurement error in SNP-exposure associations [33], the F-statistic to examine the strength of the genetic instrument [46], Cochran’s Q statistic for heterogeneity between the MR estimates for each SNP [47], and PhenoScanner was used to assess pleiotropy of the genetic instruments [48]. As sensitivity analyses, we used the MR residual sum and outlier (MR-PRESSO) to investigate the role of SNP outliers [49]. To assess pleiotropy, we used the weighted median and contamination mixture methods [50].

To explore relationships between body fat and fitness, we conducted a bi-directional MR of genetically predicted fitness on fat mass and vice versa using our genetic instrument for fitness and an instrument for total fat mass based on a GWAS of UK Biobank participants (*N* = 330,762 participants of European ancestry), derived from bioelectrical impedance measurements at study baseline [51]. We also conducted multivariable MR (MVMR) analyses to assess the effect of fitness on cancer risk, after accounting for genetically predicted fat mass and height [45].

### Statistical software

Observational analyses were performed using Stata version 16.1 (Stata Corporation, College Station, TX, USA). MR analyses were performed using the *TwoSampleMR* and *MendelianRandomisation* R packages [52, 53] and figures were plotted in R version 3.6.3. All tests of significance were two-sided, and *P* < 0.05 were considered statistically significant. Results are presented in accordance with the STROBE checklist [54].

## Results

### Observational analysis

After a median of 11 years of follow-up, 1586 prostate cancers, 1093 breast cancers, 811 colorectal cancers, 480 lung cancers, 184 endometrial cancers, and 136 ovarian cancers were diagnosed. Participant characteristics by age-adjusted and sex-specific fitness tertiles are provided in Table 1 for fitness scaled by total-body mass and Supplementary Table 2 for fitness scaled by fat-free mass. Fitness was higher in men compared to women, and those in the middle and higher fitness tertiles had better measures of adiposity, socioeconomic status, and cardiometabolic health than those in the lower fitness tertile.

**Table 1:** Participant characteristics by age-adjusted and sex-specific cardiorespiratory fitness (VO_2_max per kg total-body mass) tertiles.

		Women			Men	
	Lower fitness	Mid fitness	Higher fitness	Lower fitness	Mid fitness	Higher fitness
*N*	12,791	12,790	12,786	11,404	11,402	11,399
Age (y)	57 ± 8	57 ± 8	57 ± 8	58 ± 8	58 ± 8	58 ± 8
Height (m)	1.63 ± 0.06	1.63 ± 0.06	1.63 ± 0.06	1.76 ± 0.07	1.76 ± 0.07	1.76 ± 0.07
Total body mass (kg)	79.6 ± 14.8	69.5 ± 10.0	62.6 ± 8.2	94.4 ± 14.2	84.6 ± 10.6	77.3 ± 9.8
Fat-free mass (kg)	46.5 ± 5.4	44.0 ± 4.2	42.6 ± 3.9	66.9 ± 7.8	63.2 ± 6.9	60.6 ± 6.5
BMI (kg⋅m^−2^)	30.1 ± 5.3	26.2 ± 3.5	23.5 ± 2.8	30.3 ± 4.1	27.3 ± 2.9	25.1 ± 2.7
VO_2_max_tbm_(ml⋅min^−1^⋅kg^−1^)	19.7 ± 3.2	25.1 ± 1.9	31.4 ± 4.7	26.0 ± 2.9	31.7 ± 2.0	38.4 ± 4.2
VO_2_max_ffm_ (ml⋅min^−1^⋅kg^−1^)	33.4 ± 5.8	39.5 ± 3.8	45.8 ± 6.2	36.5 ± 4.0	42.4 ± 3.1	48.8 ± 4.8
Red meat consumption	0.8 ± 0.5	0.8 ± 0.5	0.7 ± 0.5	1.1 ± 0.6	1.0 ± 0.6	0.9 ± 0.6
Fish consumption						
Never	10.7% (1363)	9.1% (1161)	8.3% (1060)	11.8% (1343)	11.1% (1265)	8.8% (1006)
At most 1 per week	33.0% (4226)	32.2% (4114)	31.4% (4013)	36.4% (4148)	33.9% (3868)	32.3% (3687)
2 or more per week	55.6% (7116)	58.1% (7431)	60.0% (7674)	50.8% (5794)	54.4% (6202)	58.3% (6646)
Missing	0.7% (86)	0.7% (84)	0.3% (39)	1.0% (119)	0.6% (67)	0.5% (60)
Fruit & vegetable consumption						
Never	17.4% (2232)	15.4% (1974)	12.8% (1639)	24.9% (2834)	23.8% (2713)	19.3% (2199)
At most 1 per week	29.0% (3707)	28.5% (3643)	26.2% (3355)	33.5% (3816)	32.1% (3663)	31.8% (3626)
2 or more per week	53.2% (6804)	55.9% (7144)	60.8% (7772)	41.2% (4697)	43.8% (4998)	48.7% (5549)
Missing	0.4% (48)	0.2% (29)	0.2% (20)	0.5% (57)	0.2% (28)	0.2% (25)
Salt addition to meals						
Never/rarely	58.0% (7418)	58.3% (7455)	59.0% (7547)	52.8% (6022)	56.1% (6395)	60.2% (6864)
Sometimes	26.9% (3443)	27.4% (3510)	27.3% (3489)	29.3% (3343)	27.9% (3186)	25.7% (2930)
Usually/always	14.8% (1892)	14.1% (1800)	13.6% (1733)	17.5% (1994)	15.8% (1806)	13.9% (1588)
Missing	0.3% (38)	0.2% (25)	0.1% (17)	0.4% (45)	0.1% (15)	0.1% (17)
Alcohol consumption						
Never or previous	11.3% (1439)	8.2% (1055)	6.4% (812)	6.9% (785)	5.7% (648)	5.3% (599)
At most 2 per week	59.5% (7615)	52.8% (6755)	47.1% (6021)	46.4% (5286)	41.6% (4743)	39.0% (4442)
3 or more per week	28.9% (3697)	38.7% (4947)	46.4% (5930)	46.3% (5282)	52.5% (5990)	55.6% (6340)
Missing	0.3% (40)	0.3% (33)	0.2% (23)	0.4% (51)	0.2% (21)	0.2% (18)
Smoking status						
Never	63.7% (8147)	61.5% (7869)	59.7% (7635)	48.3% (5511)	51.5% (5869)	55.5% (6326)
Previous	29.0% (3704)	31.2% (3987)	32.6% (4173)	40.6% (4625)	38.2% (4357)	34.4% (3917)
Current	6.7% (858)	6.9% (879)	7.3% (932)	10.3% (1180)	9.8% (1121)	9.7% (1105)
Missing	0.6% (82)	0.4% (55)	0.4% (46)	0.8% (88)	0.5% (55)	0.4% (51)
Townsend deprivation index	−1.0 ± 3.0	−1.4 ± 2.8	−1.5 ± 2.8	−1.0 ± 3.1	−1.4 ± 2.9	−1.5 ± 2.9
Education						
No qualification	14.4% (1841)	11.0% (1413)	8.3% (1059)	14.8% (1684)	12.1% (1378)	9.4% (1072)
Any other qualification	53.8% (6885)	51.4% (6577)	46.3% (5925)	52.0% (5929)	48.9% (5579)	42.6% (4857)
Degree level or above	30.4% (3887)	36.6% (4678)	44.8% (5724)	31.7% (3618)	38.0% (4336)	47.3% (5391)
Missing	1.4% (178)	1.0% (122)	0.6% (78)	1.5% (173)	1.0% (109)	0.7% (79)
Employment						
Unemployed	9.9% (1265)	7.9% (1011)	8.3% (1061)	7.9% (903)	5.7% (651)	4.9% (557)
Employed	52.8% (6752)	55.9% (7147)	57.5% (7355)	56.7% (6467)	60.3% (6873)	61.3% (6992)
Retired	36.6% (4684)	35.7% (4572)	33.8% (4320)	34.5% (3939)	33.4% (3813)	33.3% (3794)
Missing	0.7% (90)	0.5% (60)	0.4% (50)	0.8% (95)	0.6% (65)	0.5% (56)
Race						
Asian or Asian British	3.3% (425)	2.4% (303)	1.6% (201)	3.5% (400)	3.6% (413)	2.4% (269)
Black or Black British	5.2% (670)	2.2% (281)	0.9% (117)	3.5% (398)	2.2% (254)	1.2% (138)
Mixed	1.0% (126)	0.9% (112)	0.9% (117)	0.7% (78)	0.7% (75)	0.7% (80)
Other	1.9% (243)	1.9% (240)	1.8% (234)	1.6% (180)	1.5% (174)	1.6% (180)
White	87.8% (11,235)	92.1% (11784)	94.3% (12,062)	89.9% (10,251)	91.3% (10,415)	93.6% (10,673)
Missing	0.7% (92)	0.5% (70)	0.4% (55)	0.9% (97)	0.6% (71)	0.5% (59)
Hypertension						
Not hypertensive	39.9% (5109)	56.2% (7193)	67.6% (8647)	26.2% (2987)	41.3% (4707)	55.4% (6316)
Hypertensive	60.1% (7682)	43.8% (5597)	32.4% (4139)	73.8% (8417)	58.7% (6695)	44.6% (5083)
Diabetes						
Not diabetic	93.3% (11,940)	97.3% (12,446)	98.6% (12,603)	87.5% (9980)	94.7% (10,801)	97.1% (11,067)
Diabetic	6.3% (805)	2.5% (316)	1.3% (167)	12.1% (1376)	5.1% (586)	2.8% (316)
Missing	0.4% (46)	0.2% (28)	0.1% (16)	0.4% (48)	0.1% (15)	0.1% (16)
Colorectal cancer	1.0% (133)	0.9% (112)	0.8% (98)	1.5% (169)	1.5% (173)	1.1% (126)
Colon	0.8% (103)	0.7% (93)	0.6% (74)	1.0% (119)	1.1% (122)	0.8% (87)
Rectal	0.4% (45)	0.3% (35)	0.3% (41)	0.7% (85)	0.7% (78)	0.6% (64)
Lung cancer	0.6% (77)	0.6% (71)	0.6% (82)	0.9% (103)	0.7% (76)	0.6% (71)
Breast cancer	3.2% (410)	2.8% (352)	2.6% (331)			
Endometrial cancer	0.7% (93)	0.4% (54)	0.3% (37)			
Ovarian cancer	0.3% (44)	0.3% (36)	0.4% (56)			
Prostate cancer				4.2% (481)	5.1% (579)	4.6% (526)

**Fig. 1:**
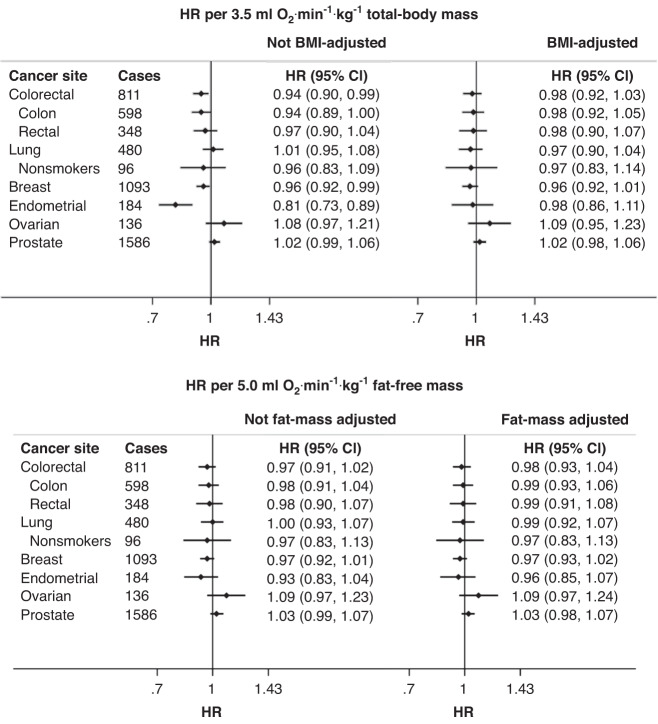
Associations of cardiorespiratory respiratory fitness and incident cancer risk without and with body fat adjustment. HRs and 95% CIs estimated using Cox regression models adjusted for age, sex, self-reported racial/ethnic group, Townsend index of deprivation, education, employment status, smoking status, alcohol consumption, red and processed meat consumption, fish consumption, fruit and vegetable consumption, salt consumption, diabetes status, hypertension, medication use (beta blockers, calcium channel blockers, ACE inhibitors, diuretics, bronchodilators, lipid-lowering agents, iron deficiency agents, non-steroidal anti-inflammatory drugs, metformin). Female reproductive cancers (breast, endometrial, and ovarian) were additionally adjusted for age at menarche, age at menopause, parity, hormone replacement therapy usage, and oral contraceptives. Associations with and without adjustment for either continuous BMI (for models with VO_2_max scaled by total-body mass) or fat mass (for models with VO_2_max scaled by fat-free mass). ACE Angiotensin-converting enzyme, BMI body mass index, CI confidence interval, HR hazard ratio.

Observational analysis results are summarised in Fig. 1. In analyses without BMI adjustment, each 3.5 ml O_2_⋅min^−1^⋅kg^−1^ total-body mass increase (equivalent to 1 metabolic equivalent of task [MET]) in fitness was associated with a 19% reduction in endometrial cancer, 6% reduction in colorectal cancer, and 4% reduction in breast cancer. After BMI adjustment, associations were attenuated but remained directionally consistent. Where associations were detected, relationships generally appeared to be linear but with uncertainty for some cancers at the tails of the fitness distribution (Supplementary Figs. 3 and 4). When fitness was expressed per kg fat-free mass, associations with cancers were not significant. Results adjusted for regression dilution are shown in Supplementary Fig. 5.

**Fig. 2:**
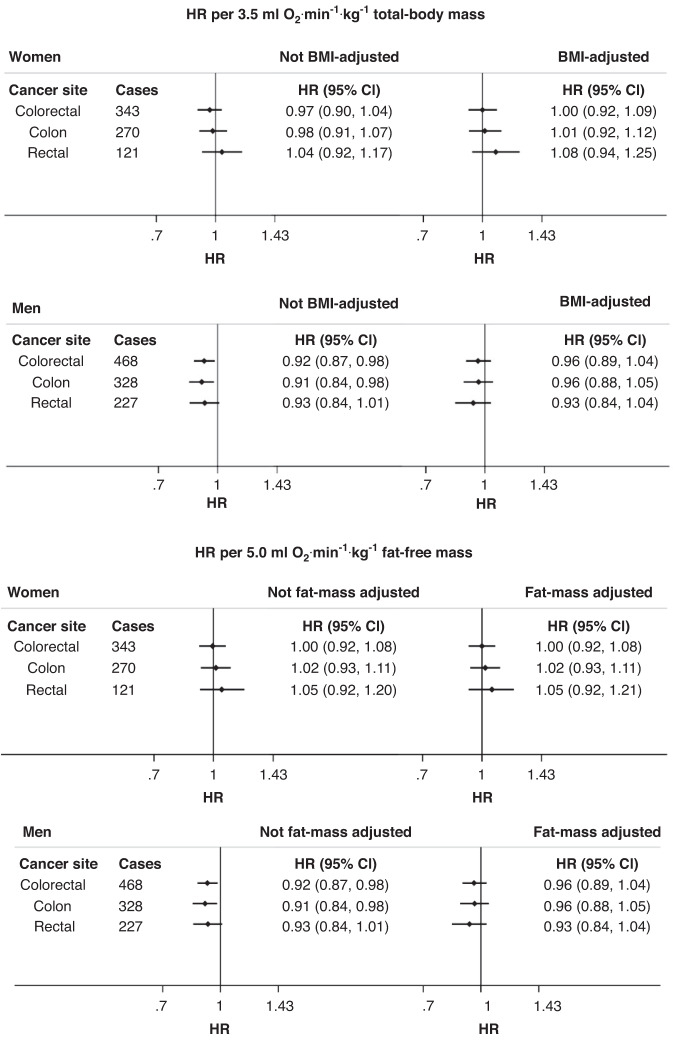
Sex-stratified associations of cardiorespiratory respiratory fitness and incident cancer risk without and with body fat adjustment. HRs and 95% CIs estimated using Cox regression models adjusted for age, sex, self-reported racial/ethnic group, Townsend index of deprivation, education, employment status, smoking status, alcohol consumption, red and processed meat consumption, fish consumption, fruit and vegetable consumption, salt consumption, diabetes status, hypertension, medication use (beta blockers, calcium channel blockers, ACE inhibitors, diuretics, bronchodilators, lipid-lowering agents, iron deficiency agents, non-steroidal anti-inflammatory drugs, metformin). Associations with and without adjustment for either continuous BMI (for models with VO_2_max scaled by total-body mass) or fat mass (for models with VO_2_max scaled by fat-free mass). ACE Angiotensin-converting enzyme, BMI body mass index, CI confidence interval, HR hazard ratio.

There was evidence of heterogeneity in the associations of fitness and colorectal cancers by sex; the relationship was inverse for men and null for women (Fig. 2 and Supplementary Fig. 4). Minimally adjusted models are available from Supplementary Table 3.

### Mendelian randomisation analyses

**Fig. 3:**
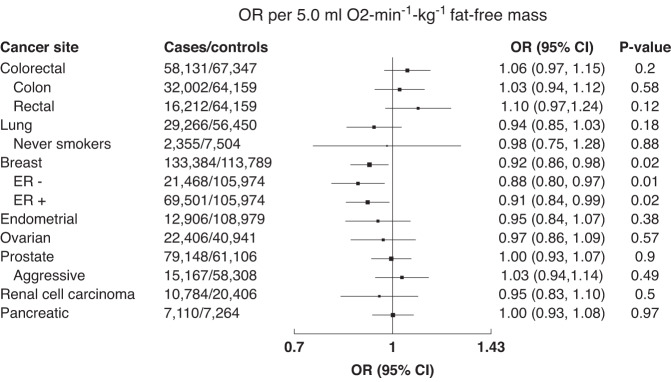
Associations of genetically predicted cardiorespiratory respiratory fitness and cancer risk. Associations were estimated using the inverse variance weighted method. CI confidence interval, ER estrogen receptor, OR odds ratio.

Higher levels of genetically predicted fitness were associated with a lower risk of breast cancer (OR per 5.0 ml O_2_⋅min^−1^⋅kg^−1^ fat-free mass = 0.92, 95% CI: 0.86–0.98; *P* = 0.02), including ER+ (0.91, 0.84–0.99; *P* = 0.02) and ER- (0.88, 0.80–0.97; *P* = 0.01) subtypes, but was not significantly associated with any other cancer site (Fig. 3). There was also no evidence of an association with colorectal cancer after stratification by sex and site (Supplementary Tables 4 and 5). There was significant heterogeneity in the MR estimates for the SNPs for each cancer site (Cochran’s Q *P* < 0.05), except for associations with lung cancer for never smokers (*P* = 0.13), aggressive prostate cancer (*P* = 0.17) and renal cancer (*P* = 0.09).

In MR sensitivity analyses, the relationships between fitness and breast cancer were directionally consistent in comparison with the primary MR analysis (Supplementary Table 6). There was evidence of an inverse association between fitness and lung cancer using the weighted median method (0.85, 0.74–0.98; *P* = 0.02) and a positive association with pancreatic cancer using the contamination mixture method (1.09, 1.03–1.14; *P* = 0.03) (Supplementary Table 6). Radial plots also did not indicate any strong influence of outliers on the MR results (Supplementary Fig. 6). The likelihood of bias due to weak instruments was low (F-statistic > 10 for all SNPs). There was evidence of moderate levels of measurement error (I^2^_GX_ = 0.52–0.65), indicating reduced reliability of Egger results, therefore we do not include Egger estimates [55]. Using PhenoScanner, 742 traits were linked to SNPs for fitness (*P* < 5 × 10^−8^), particularly pulse rate (Supplementary Fig. 7).

The bi-directional MR analysis indicated that genetically instrumented fat mass had a strong inverse association with fitness (OR per 0.5 SD increase = 0.61, 0.52–0.71; *P* < 0.001), but a weaker inverse relationship of fitness with fat mass (OR per 5 ml O_2_⋅min^−1^⋅kg^−1^ fat-free mass = 0.96, 0.92–1.01; *P* = 0.08). In MVMR analyses, associations with breast cancer were attenuated after adjustment for fat mass and height. While associations with lung cancer became statistically significant (0.90, 0.84–0.96; *P* = 0.002), although remained null for never smokers (Table 2).

**Table 2:** Genetic associations of cardiorespiratory respiratory fitness and cancer risk after accounting for fat mass and height.

Cancer site	OR per 5.0 ml O_2_⋅min^−1^⋅kg^−1^ fat-free mass (95% CI)	*P*-value
Colorectal	1.03 (0.97, 1.10)	0.29
Colon	1.00 (0.94, 1.07)	0.94
Rectal	1.04 (0.96, 1.13)	0.31
Lung	**0.90 (0.84, 0.96)**	**0.002**
Never smokers	0.98 (0.82, 1.17)	0.83
Breast	0.98 (0.93, 1.03)	0.39
ER −	0.95 (0.89, 1.02)	0.14
ER +	0.97 (0.92, 1.03)	0.36
Endometrial	0.97 (0.89, 1.06)	0.24
Ovarian	1.00 (0.92, 1.08)	0.99
Prostate	1.01 (0.95, 1.08)	0.66
Aggressive	1.01 (0.93, 1.10)	0.80
Renal cell carcinoma	0.86 (0.73, 1.01)	0.07
Pancreatic	1.04 (0.92, 1.17)	0.54

Risk estimates based on multivariable Mendelian randomisation. Risk estimates *p* < 0.05 are in bold.

*CI* confidence interval, *ER* estrogen receptor, *OR* odds ratio.

## Discussion

This study used both observational and MR methods to examine the relationship between cardiorespiratory fitness and incident cancer risk, providing the first evidence that higher fitness levels may reduce risks of breast cancer. In observational analysis only, we report additional inverse associations between VO_2_max scaled to total body mass and risks of colorectal and endometrial cancer. However, associations with all three cancer sites were attenuated after accounting for adiposity. Observational associations between cancer and VO_2_max scaled to fat-free mass were not statistically significant.

Previous observational analyses have reported inverse associations between fitness and colorectal and lung cancer. We did not observe an association with lung cancer and the inverse association between VO_2_max scaled to total body mass and colorectal cancer was attenuated after accounting for BMI [12–15]. Our results may differ from these previous studies due to differences in population sampling, fitness assessment, and fitness estimation approaches. For example, cycle ergometer-based fitness estimates may differ from treadmill-based estimates due to differences in load bearing and motion artefact [15, 18, 20]. The UK Biobank fitness test was also relatively light intensity, which enabled more participants to be assessed. Thus, our analysis likely characterises a wider variety of lower-fitness individuals than previous studies which used more strenuous tests. Previous estimates using UK Biobank data had shorter duration of follow-up (median 5 years) and used fewer exercise test data, which will reduce the precision of risk estimates.

Previous MR studies based on up to five SNPs have reported inverse associations between genetically predicted physical activity levels and risks of breast, colorectal and aggressive prostate cancer [56, 57]. However, current estimates suggest that GWAS significant polymorphisms explain a very limited proportion of phenotypic physical activity (e.g., 0.06% for overall physical activity) [58]. The small number of SNPs increase the influence of possibly invalid variants within the instrument, and the instrument has a bidirectional association with BMI [58]. Fitness is a trait that reflects both input from genetics and physical activity behaviours. The genetic instrument for fitness used in the present study likely encompasses both past and current levels as well as the capacity to participate in physical activity [2, 3]. This instrument explains 1.2% of the variation in observed fitness levels, increasing the reliability of risk estimates. Future work examining the relative importance of the different constituents of genetic fitness may help to clarify whether the null relationships that we report for fitness on colorectal and aggressive prostate cancer risk are indicative of the greater relative importance of physical activity behaviours or are partially reflective of the methodological limitations discussed above.

The role of adiposity in fitness is complex and not fully understood. Higher adiposity is associated with impaired physical performance, relating reduced muscle oxygen uptake, lower cardiac efficiency, neuromuscular dysfunction, and increased cancer risk [59–63]. Higher levels of physical activity are important for weight maintenance and increasing fitness, and higher fitness may reduce some of the harmful cardiometabolic effects of obesity [64]. Differences between the associations of fitness and cancer by scaling are likely driven by the different components of fitness, as VO_2_max_tbm_ has a strong inverse correlation with body size and adiposity [27]. However, the complex interplay of adiposity, fitness and cancer might mean that accounting for adiposity for models of cardiorespiratory fitness could lead to an over-adjustment of risk estimates, but these relationships are difficult to disentangle. Relationships between fitness and all-cause, cancer, and cardiovascular mortality outcomes has stronger evidence for independence of associations with adiposity [10, 64–66]. Future work with longer durations of follow-up will improve power to investigate whether there are differential risk associations by BMI classification.

These analyses have several strengths. This study is the first to use genetically instrumented fitness to evaluate possible causal relationships between fitness and cancer risk. The UK Biobank is the largest sample currently available with measured cardiorespiratory fitness, maximising power to assess associations across a broad range of cancer sites, the majority of which have not been previously investigated. Our independently validated novel framework to estimate fitness harmonised the UK Biobank test protocols and calibrated these data to a maximal exercise test to estimate VO_2_max. This estimation framework also incorporated multiple heart rate measurements to reduce measurement error, with high temporal agreement (regression dilution ratio=0.79) over approximately a 2.8 year period for greater precision in risk estimates [43]. Further, the baseline assessment collected data across a wide range of lifestyle, medical and anthropometric factors, enabling thorough adjustment for possible confounders.

Our study has limitations. This analysis is not a randomised controlled trial and therefore we are not able to fully assess causality. In MR analysis we cannot exclude the possibility of genetic confounding or horizontal pleiotropy [67]. The genetic instrument for VO_2_max_tbm_ was not available for comparison with our observational analysis. The genetic instrument also included resting heart rate information; therefore, our results may be partially driven by genetic associations with resting heart rate. Given the strong a priori evidence and mechanistic plausibility of associations between fitness and cancer risk we have not included correction for multiple testing [18–20], however, we cannot exclude the possibility of chance findings. The UK Biobank participants are predominantly of White European ancestry and are healthier than the underlying sampling population, therefore risk estimates may not be generalisable to some other populations, including “high-risk” participants who did not undergo the fitness assessment. The fitness test was also submaximal, which may increase measurement error, and previous studies have noted larger magnitudes of associations with health outcomes using maximal fitness tests [11, 15].

In summary, we provide evidence that higher fitness levels may reduce risks of endometrial, colorectal, and breast cancer. The role of adiposity in mediating the relationship between fitness and cancer risk is not fully understood, and further research is needed to explore this complex relationship. Aiming to increase fitness, including via changes in body composition, may be an effective strategy to reduce risk of some cancer sites.

### Supplementary information

Supplementary Materials and Methods

Appendix 1

## Data Availability

The UK Biobank is an open-access resource and bona fide researchers can apply to use the UK Biobank dataset by registering and applying at http://ukbiobank.ac.uk/register-apply/. Further information is available from the corresponding author upon request. Fitness estimates for each participant have been returned to UK Biobank for download by registered researchers. For genetic cancer data, summary GWAS statistics are publicly available for breast (https://bcac.ccge.medschl.cam.ac.uk/), endometrial (https://www.ebi.ac.uk/gwas/studies/GCST006464), ovarian (https://ocac.ccge.medschl.cam.ac.uk) and prostate (overall only) (http://practical.icr.ac.uk/). Data for renal cell carcinoma and pancreatic cancer were accessed via dbGaP; Study Accession: phs000206.v3.p2 and phs000648.v1.p1; project reference 9314 (https://www.ncbi.nlm.nih.gov/gap/). Summary genetic data for aggressive prostate cancer (http://practical.icr.ac.uk/), lung cancer (https://ilcco.iarc.fr/) and colorectal cancer (https://research.fredhutch.org/peters/en/genetics-and-epidemiology-of-colorectal-cancer-consortium.html) are not currently publicly available but may be made available upon application, see respective websites for details.

## Code availability

Analyses were performed using Stata version 16.1 (Stata Corporation, College Station, TX, USA) and R (version 4.3.1) the code is available upon reasonable request.

## References

[1] Moore SC, Lee IM, Weiderpass E, Campbell PT, Sampson JN, Kitahara CM (2016). Association of leisure-time physical activity with risk of 26 types of cancer in 1.44 Million adults. JAMA Intern Med.

[2] Sarzynski MA, Ghosh S, Bouchard C (2017). Genomic and transcriptomic predictors of response levels to endurance exercise training. J Physiol.

[3] Kim DS, Wheeler MT, Ashley EA (2022). The genetics of human performance. Nat Rev Genet.

[4] Bouchard C, An P, Rice T, Skinner JS, Wilmore JH, Gagnon J (1999). Familial aggregation of VO2max response to exercise training: results from the HERITAGE Family Study. J Appl Physiol.

[5] Al-Mallah MH, Sakr S, Al-Qunaibet A (2018). Cardiorespiratory fitness and cardiovascular disease prevention: an update. Curr Atheroscler Rep.

[6] Kaze AD, Agoons DD, Santhanam P, Erqou S, Ahima RS, Echouffo-Tcheugui JB (2022). Correlates of cardiorespiratory fitness among overweight or obese individuals with type 2 diabetes. BMJ Open Diabetes Res Care.

[7] LaMonte MJ, Eisenman PA, Adams TD, Shultz BB, Ainsworth BE, Yanowitz FG (2000). Cardiorespiratory fitness and coronary heart disease risk factors: the LDS Hospital Fitness Institute cohort. Circulation..

[8] Arsenault BJ, Lachance D, Lemieux I, Alméras N, Tremblay A, Bouchard C (2007). Visceral adipose tissue accumulation, cardiorespiratory fitness, and features of the metabolic syndrome. Arch Intern Med.

[9] Han M, Qie R, Shi X, Yang Y, Lu J, Hu F, et al. Cardiorespiratory fitness and mortality from all causes, cardiovascular disease and cancer: dose–response meta-analysis of cohort studies. Br J Sports Med [Internet]. 2022. [cited 2022 May 13]; Available from: https://bjsm.bmj.com/content/early/2022/01/12/bjsports-2021-104876. 10.1136/bjsports-2021-104876 35022163

[10] Schmid D, Leitzmann MF (2015). Cardiorespiratory fitness as predictor of cancer mortality: a systematic review and meta-analysis. Ann Oncol.

[11] Laukkanen JA, Isiozor NM, Kunutsor SK (2022). Objectively assessed cardiorespiratory fitness and all-cause mortality risk: an updated meta-analysis of 37 cohort studies involving 2,258,029 participants. Mayo Clin Proc.

[12] Marshall CH, Al-Mallah MH, Dardari Z, Brawner CA, Lamerato LE, Keteyian SJ (2019). Cardiorespiratory fitness and incident lung and colorectal cancer in men and women: results from the Henry Ford Exercise Testing (FIT) cohort. Cancer..

[13] Pozuelo-Carrascosa DP, Alvarez-Bueno C, Cavero-Redondo I, Morais S, Lee IM, Martínez-Vizcaíno V (2019). Cardiorespiratory fitness and site-specific risk of cancer in men: a systematic review and meta-analysis. Eur J Cancer.

[14] Steell L, Ho FK, Sillars A, Petermann-Rocha F, Li H, Lyall DM (2019). Dose-response associations of cardiorespiratory fitness with all-cause mortality and incidence and mortality of cancer and cardiovascular and respiratory diseases: the UK Biobank cohort study. Br J Sports Med.

[15] Lakoski SG, Willis BL, Barlow CE, Leonard D, Gao A, Radford NB (2015). Midlife cardiorespiratory fitness, incident cancer, and survival after cancer in men: the cooper center longitudinal study. JAMA Oncol.

[16] Hillreiner A, Baumeister SE, Sedlmeier AM, Finger JD, Schlitt HJ, Leitzmann MF (2020). Association between cardiorespiratory fitness and colorectal cancer in the UK Biobank. Eur J Epidemiol.

[17] Ekblom-Bak E, Bojsen-Møller E, Wallin P, Paulsson S, Lindwall M, Rundqvist H (2023). Association between cardiorespiratory fitness and cancer incidence and cancer-specific mortality of colon, lung, and prostate cancer among Swedish Men. JAMA Netw Open.

[18] Vainshelboim B, Müller J, Lima RM, Nead KT, Chester C, Chan K (2017). Cardiorespiratory fitness and cancer incidence in men. Ann Epidemiol.

[19] Kunutsor SK, Voutilainen A, Laukkanen JA (2021). Cardiorespiratory fitness is not associated with reduced risk of prostate cancer: a cohort study and review of the literature. Eur J Clin Invest.

[20] Byun W, Sui X, Hébert JR, Church TS, Lee IM, Matthews CE (2011). Cardiorespiratory fitness and risk of prostate cancer: findings from the Aerobics Center Longitudinal Study. Cancer Epidemiol.

[21] Cai L, Gonzales T, Wheeler E, Kerrison N, Day F, Langenberg C, et al. Causal associations between cardiorespiratory fitness and type 2 diabetes. Nat Commun. 2023;14:3904. 10.1038/s41467-023-38234-w PMC10318084 37400433

[22] Lawlor DA, Tilling K, Davey Smith G (2016). Triangulation in aetiological epidemiology. Int J Epidemiol.

[23] UK Biobank: Protocol for a large-scale prospective epidemiological resource [Internet]. 2007. Available from: https://www.ukbiobank.ac.uk/media/gnkeyh2q/study-rationale.pdf.

[24] UK Biobank cardio assessment manual: Version 1.0 [Internet]. 2011. Available from: http://biobank.ctsu.ox.ac.uk/crystal/docs/Cardio.pdf.

[25] Imboden MT, Kaminsky LA, Peterman JE, Hutzler HL, Whaley MH, Fleenor BS (2020). Cardiorespiratory fitness normalized to fat-free mass and mortality risk. Med Sci Sports Exerc.

[26] Osman AF, Mehra MR, Lavie CJ, Nunez E, Milani RV (2000). The incremental prognostic importance of body fat adjusted peak oxygen consumption in chronic heart failure. J Am Coll Cardiol.

[27] Zhou N (2021). Assessment of aerobic exercise capacity in obesity, which expression of oxygen uptake is the best?. Sports Med Health Sci.

[28] Bowden J, Spiller W, Del Greco MF, Sheehan N, Thompson J, Minelli C (2018). Improving the visualization, interpretation and analysis of two-sample summary data Mendelian randomization via the Radial plot and Radial regression. Int J Epidemiol.

[29] Kokkinos P, Myers J, Franklin B, Narayan P, Lavie CJ, Faselis C (2018). Cardiorespiratory fitness and health outcomes: a call to standardize fitness categories. Mayo Clin Proc.

[30] Haycock PC, Burgess S, Wade KH, Bowden J, Relton C, Davey Smith G (2016). Best (but oft-forgotten) practices: the design, analysis, and interpretation of Mendelian randomization studies1. Am J Clin Nutr.

[31] Michailidou K, Lindström S, Dennis J, Beesley J, Hui S, Kar S (2017). Association analysis identifies 65 new breast cancer risk loci. Nature..

[32] Zhang H, Ahearn TU, Lecarpentier J, Barnes D, Beesley J, Qi G (2020). Genome-wide association study identifies 32 novel breast cancer susceptibility loci from overall and subtype-specific analyses. Nat Genet.

[33] Schumacher FR, Al Olama AA, Berndt SI, Benlloch S, Ahmed M, Saunders EJ (2018). Association analyses of more than 140,000 men identify 63 new prostate cancer susceptibility loci. Nat Genet.

[34] O’Mara TA, Glubb DM, Amant F, Annibali D, Ashton K, Attia J (2018). Identification of nine new susceptibility loci for endometrial cancer. Nat Commun.

[35] Phelan CM, Kuchenbaecker KB, Tyrer JP, Kar SP, Lawrenson K, Winham SJ (2017). Identification of 12 new susceptibility loci for different histotypes of epithelial ovarian cancer. Nat Genet.

[36] McKay JD, Hung RJ, Han Y, Zong X, Carreras-Torres R, Christiani DC (2017). Large-scale association analysis identifies new lung cancer susceptibility loci and heterogeneity in genetic susceptibility across histological subtypes. Nat Genet.

[37] Huyghe JR, Bien SA, Harrison TA, Kang HM, Chen S, Schmit SL (2019). Discovery of common and rare genetic risk variants for colorectal cancer. Nat Genet.

[38] Huyghe JR, Harrison TA, Bien SA, Hampel H, Figueiredo JC, Schmit SL (2021). Genetic architectures of proximal and distal colorectal cancer are partly distinct. Gut..

[39] Scelo G, Purdue MP, Brown KM, Johansson M, Wang Z, Eckel-Passow JE (2017). Genome-wide association study identifies multiple risk loci for renal cell carcinoma. Nat Commun.

[40] Childs EJ, Mocci E, Campa D, Bracci PM, Gallinger S, Goggins M (2015). Common variation at 2p13.3, 3q29, 7p13 and 17q25.1 associated with susceptibility to pancreatic cancer. Nat Genet..

[41] Petersen GM, Amundadottir L, Fuchs CS, Kraft P, Stolzenberg-Solomon RZ, Jacobs KB (2010). A genome-wide association study identifies pancreatic cancer susceptibility loci on chromosomes 13q22.1, 1q32.1 and 5p15.33. Nat Genet..

[42] Amundadottir L, Kraft P, Stolzenberg-Solomon RZ, Fuchs CS, Petersen GM, Arslan AA (2009). Genome-wide association study identifies variants in the ABO locus associated with susceptibility to pancreatic cancer. Nat Genet.

[43] Gonzales TI, Westgate K, Strain T, Hollidge S, Jeon J, Christensen DL (2021). Cardiorespiratory fitness assessment using risk-stratified exercise testing and dose-response relationships with disease outcomes. Sci Rep.

[44] Keogh RH, White IR (2014). A toolkit for measurement error correction, with a focus on nutritional epidemiology. Stat Med.

[45] Burgess S, Bowden J. Integrating summarized data from multiple genetic variants in Mendelian randomization: Bias and coverage properties of inverse-variance weighted methods [Internet]. arXiv; 2015 [cited 2022 May 13]. Available from: http://arxiv.org/abs/1512.04486.

[46] Burgess S, Thompson SG, CHD CRP (2011). Genetics collaboration. Avoiding bias from weak instruments in Mendelian randomization studies. Int J Epidemiol.

[47] Burgess S, Bowden J, Fall T, Ingelsson E, Thompson SG (2017). Sensitivity analyses for robust causal inference from Mendelian randomization analyses with multiple genetic variants. Epidemiol Camb Mass.

[48] Kamat MA, Blackshaw JA, Young R, Surendran P, Burgess S, Danesh J (2019). PhenoScanner V2: an expanded tool for searching human genotype-phenotype associations. Bioinforma Oxf Engl.

[49] Verbanck M, Chen CY, Neale B, Do R (2018). Detection of widespread horizontal pleiotropy in causal relationships inferred from Mendelian randomization between complex traits and diseases. Nat Genet.

[50] Bowden J, Davey, Smith G, Haycock PC, Burgess S (2016). Consistent estimation in Mendelian randomization with some invalid instruments using a weighted median estimator. Genet Epidemiol.

[51] Neale lab [Internet]. [cited 2022 Aug 2]. UK Biobank. Available from: http://www.nealelab.is/uk-biobank.

[52] Hemani G, Zheng J, Elsworth B, Wade KH, Haberland V, Baird D (2018). The MR-Base platform supports systematic causal inference across the human phenome. eLife..

[53] Yavorska OO, Burgess S (2017). MendelianRandomization: an R package for performing Mendelian randomization analyses using summarized data. Int J Epidemiol.

[54] Mansournia MA, Collins GS, Nielsen RO, Nazemipour M, Jewell NP, Altman DG (2021). A checklist for statistical assessment of medical papers: the CHAMP statement. Br J Sports Med.

[55] Bowden J, Del Greco MF, Minelli C, Davey Smith G, Sheehan NA, Thompson JR (2016). Assessing the suitability of summary data for two-sample Mendelian randomization analyses using MR-Egger regression: the role of the I2 statistic. Int J Epidemiol.

[56] Kazmi N, Haycock P, Tsilidis K, Lynch BM, Truong T (2020). PRACTICAL consortium, CRUK, BPC3, CAPS, PEGASUS, et al. Appraising causal relationships of dietary, nutritional and physical-activity exposures with overall and aggressive prostate cancer: two-sample Mendelian-randomization study based on 79,148 prostate-cancer cases and 61,106 controls. Int J Epidemiol.

[57] Papadimitriou N, Dimou N, Tsilidis KK, Banbury B, Martin RM, Lewis SJ (2020). Physical activity and risks of breast and colorectal cancer: a Mendelian randomisation analysis. Nat Commun.

[58] Doherty A, Smith-Byrne K, Ferreira T, Holmes MV, Holmes C, Pulit SL (2018). GWAS identifies 14 loci for device-measured physical activity and sleep duration. Nat Commun.

[59] Wearing SC, Hennig EM, Byrne NM, Steele JR, Hills AP (2006). The impact of childhood obesity on musculoskeletal form. Obes Rev Off J Int Assoc Study Obes.

[60] Hung TH, Liao PA, Chang HH, Wang JH, Wu MC (2014). Examining the relationship between cardiorespiratory fitness and body weight status: empirical evidence from a population-based survey of adults in Taiwan. ScientificWorldJournal..

[61] Tanner CJ, Barakat HA, Dohm GL, Pories WJ, MacDonald KG, Cunningham PRG (2002). Muscle fiber type is associated with obesity and weight loss. Am J Physiol Endocrinol Metab.

[62] Norman AC, Drinkard B, McDuffie JR, Ghorbani S, Yanoff LB, Yanovski JA (2005). Influence of excess adiposity on exercise fitness and performance in overweight children and adolescents. Pediatrics..

[63] Larsson SC, Spyrou N, Mantzoros CS (2022). Body fatness associations with cancer: evidence from recent epidemiological studies and future directions. Metabolism..

[64] Barry VW, Baruth M, Beets MW, Durstine JL, Liu J, Blair SN (2014). Fitness vs. fatness on all-cause mortality: a meta-analysis. Prog Cardiovasc Dis.

[65] Farrell SW, Cortese GM, LaMonte MJ, Blair SN (2007). Cardiorespiratory fitness, different measures of adiposity, and cancer mortality in men. Obesity..

[66] Sui X, LaMonte MJ, Laditka JN, Hardin JW, Chase N, Hooker SP (2007). Cardiorespiratory fitness and adiposity as mortality predictors in older adults. JAMA J Am Med Assoc.

[67] Davies NM, Holmes MV, Smith GD (2018). Reading Mendelian randomisation studies: a guide, glossary, and checklist for clinicians. BMJ..

